# Reflective AI use and student engagement in AI-supported programming: technology acceptance, programming self-efficacy, and behavioral trace evidence

**DOI:** 10.3389/fpsyg.2026.1929887

**Published:** 2026-09-15

**Authors:** Sheng Wang, Jiabi Zhao

**Affiliations:** 1 Shanghai University of Finance and Economics Zhejiang College, Jinhua, China; 2 School of Management, Zhejiang University, Hangzhou, Zhejiang, China

**Keywords:** AI-supported programming, behavioral trace analytics, generative AI, programming self-efficacy, reflective AI use, self-regulated learning, student engagement, technology acceptance

## Abstract

**Introduction:**

AI coding assistants are increasingly used in programming education, but willingness to use them does not show whether students evaluate or learn from AI suggestions. Grounded in technology acceptance, self-regulated learning, and self-efficacy theory, this study examined reflective AI use and student engagement.

**Methods:**

The Reflective AI Acceptance Integration Framework (RAIAF) organized three independent public datasets: a survey structural equation model (*N* = 131), double human coding of 66 nonblank Critical Engagement responses from a Copilot field study, and complementary programming-behavior prediction using DTA logs (*N* = 1, 423; 40% calendar-window sample *N* = 1, 122). The DTA dataset did not measure AI use and was used only to test whether recovery-related programming traces added predictive information.

**Results:**

In the MLR survey model, technology acceptance predicted reflective use (β = 0.694, *p* < 0.001) and engagement (β = 0.694, *p* < 0.001); reflective use predicted programming self-efficacy (β = 0.519, *p* < 0.001) and engagement (β = 0.273, *p* = 0.034). None of the hypothesized indirect effects was significant. Initial text-coder agreement was limited (linear-weighted κ = 0.421; ordinal α = 0.495), and consensus-coded reflective use did not significantly predict productive engagement (*b* = 0.149, *p* = 0.202). In DTA, adding recovery features increased held-out final-success Macro-F1 from 0.725 to 0.743, but average gains in repeated and 30%–50% window analyses were negligible. Activity-defined engagement was already captured by overlapping activity indicators.

**Discussion:**

The findings distinguish acceptance from reflective use but do not establish a general mediation mechanism. Reflective use was associated with self-efficacy and engagement in the survey, whereas its text and behavioral evidence was exploratory, small, and context dependent.

## 1 Introduction

Artificial intelligence (AI) is changing the psychological conditions under which students learn programming. In AI-supported programming environments, learners can receive code suggestions, debugging hints, explanations, and automated feedback while they are solving tasks. These functions may reduce frustration and help novice programmers continue when they encounter syntax errors, failed test cases, or unclear requirements. However, programming learning is not only the production of working code. Students must also monitor the quality of external feedback, judge whether an AI suggestion is appropriate, revise errors, and transfer what they have learned to new problems. The central educational-psychological issue is therefore not simply whether AI tools are accepted, but whether students engage with AI-generated support in reflective and learning-oriented ways.

This problem is timely because generative AI changes the relation between help seeking, feedback use, and learner agency. A student may accept an AI programming assistant because it is convenient, but still copy suggestions passively. Another student may compare a suggestion with personal reasoning, identify a flaw, revise the code, and use the feedback to build confidence for later tasks. These two learners may appear similar if only tool acceptance or use frequency is measured, but they differ psychologically in metacognitive monitoring, feedback interpretation, and self-regulated learning. The present study focuses on this difference by examining how students' acceptance of AI tools, reflective AI use, programming self-efficacy, and engagement are related.

Several research traditions inform this question. Technology acceptance research explains why learners are willing to use digital tools, especially through perceived usefulness and perceived ease of use (Davis, 1989; Venkatesh et al., 2003). Student-engagement research suggests that learning involvement includes behavioral, emotional, and cognitive dimensions (Fredricks et al., 2004). Self-regulated learning and metacognition research explains how learners plan, monitor, and adjust strategies when using feedback (Zimmerman, 2002; Pintrich, 2000; Winne and Hadwin, 1998). Programming self-efficacy research further shows that confidence in programming capability is related to persistence and achievement in difficult coding tasks (Bandura, 1997; Ramalingam and Wiedenbeck, 1998). Recent work on generative AI and computing education has examined ChatGPT, GitHub Copilot, code generation, and automated feedback (Kasneci et al., 2023; Milano et al., 2023; Thorp, 2023; Noy and Zhang, 2023; Doshi and Hauser, 2024; Denny et al., 2024; Raihan et al., 2025). Together, these studies suggest a key limitation: positive attitudes toward AI tools do not reveal whether students check, revise, and internalize AI-generated suggestions.

This limitation motivates the present study. We define reflective AI use as students' active evaluation, explanation, correction, and revision of AI-supported feedback. Reflective AI use is not treated as mere frequency of tool use; rather, it is a psychologically meaningful form of feedback engagement. We also focus on programming self-efficacy, which captures students' perceived capability to complete programming tasks. The main task of this paper is to examine whether technology acceptance is associated with reflective AI use, whether reflective AI use is associated with programming self-efficacy, and whether these constructs help explain engagement in AI-supported programming. A secondary task is to examine whether reflection-related behaviors can be observed in text responses and programming traces.

To address these tasks, we organize the analyses through the Reflective AI Acceptance Integration Framework (RAIAF). As shown in Figure 1, RAIAF integrates three complementary sources of evidence. First, a survey-based structural model tests the relationships among technology acceptance, reflective use, programming self-efficacy, and learning engagement. Second, a Copilot field-study text analysis explores whether reflective use can be observed from students' open-ended Critical Engagement responses. Third, a DTA programming-log analysis constructs early-window behavioral indicators and predicts later learning outcomes. This design is intentionally transparent and conservative. It does not assume that all psychological paths must be significant. Instead, it asks where the proposed psychological mechanism is supported, where it is weak, and how far behavioral trace evidence can extend the interpretation.

**Figure 1:**
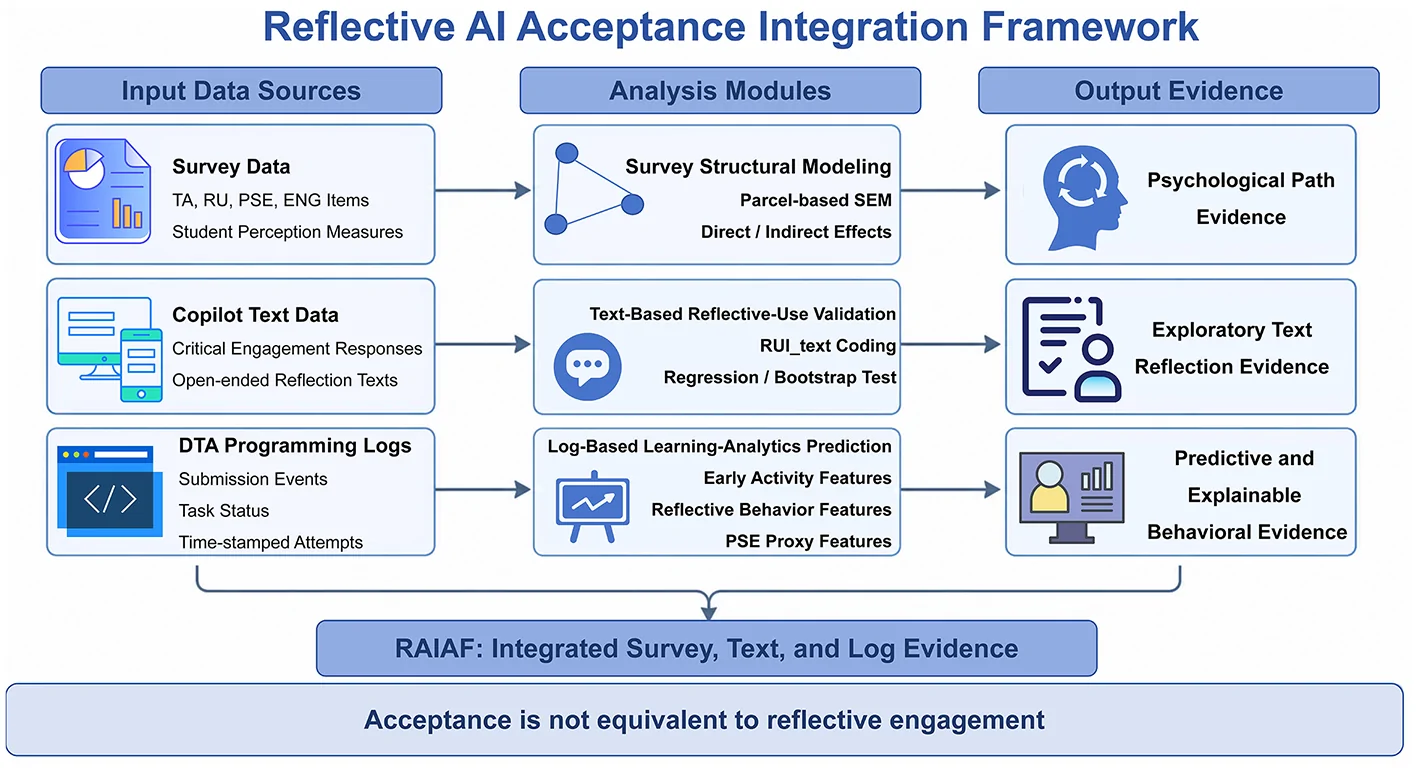
Overview of the Reflective AI Acceptance Integration Framework (RAIAF). The framework integrates survey data, Copilot text data, and DTA programming logs to examine how technology acceptance, reflective use, programming self-efficacy, and early behavioral features contribute to understanding student engagement in AI-supported programming.

The empirical results later summarized in Figures 2–4 further illustrate this logic. Technology acceptance directly predicts engagement in the survey model, whereas recovery-related DTA features provide only small and evaluation-dependent differences in final-success prediction. This contrast supports a cautious conclusion: evidence about reflective processes depends on both measurement and outcome context.

**Figure 2:**
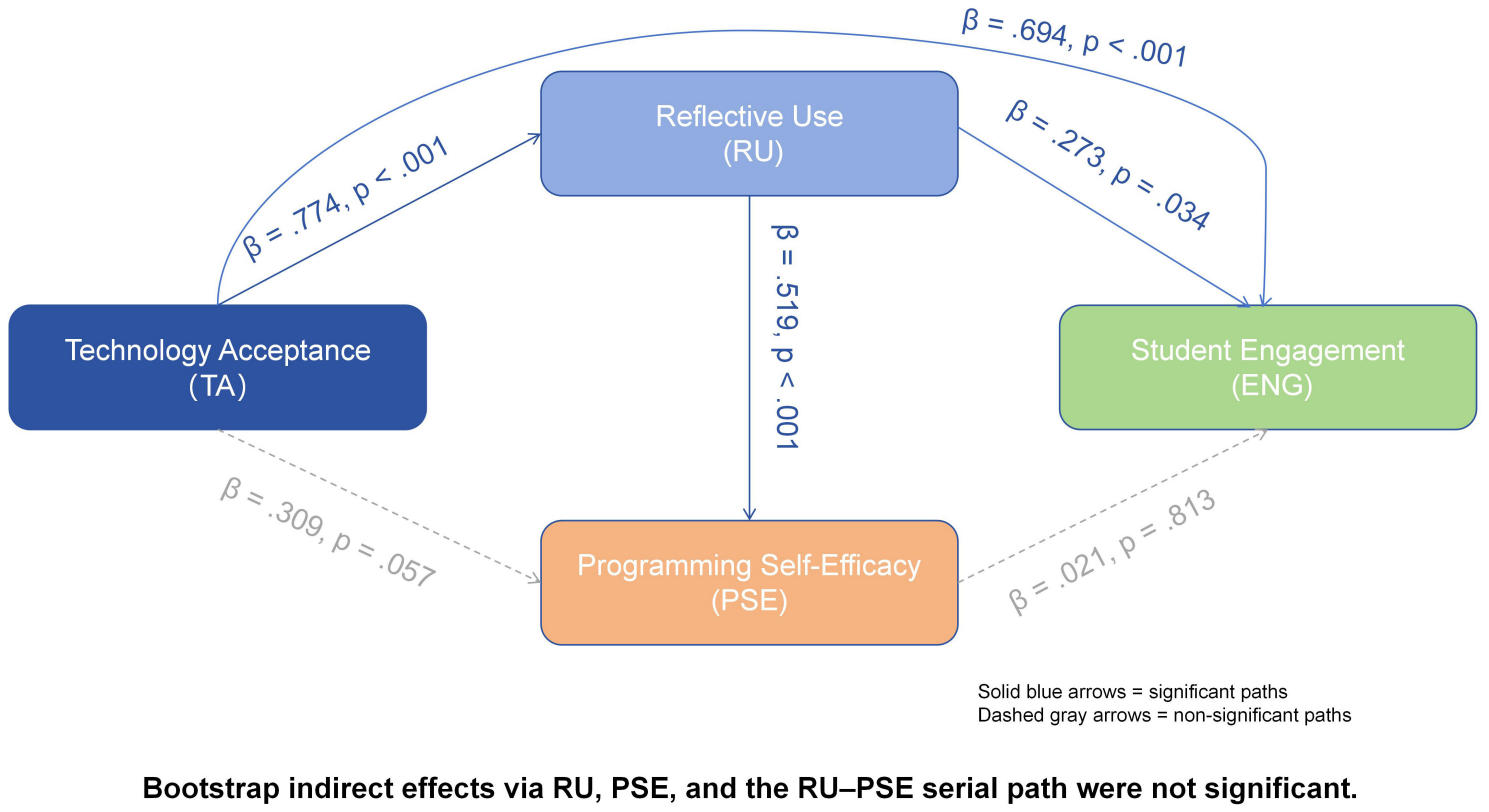
Survey-based structural path model. Solid blue arrows indicate significant MLR paths, and dashed gray arrows indicate non-significant paths. The parcel-based SEM showed CFI = 0.951, TLI = 0.934, RMSEA = 0.082, and SRMR = 0.029. Bootstrap indirect effects via RU, PSE, and the RU–PSE serial path were not statistically significant.

**Figure 3:**
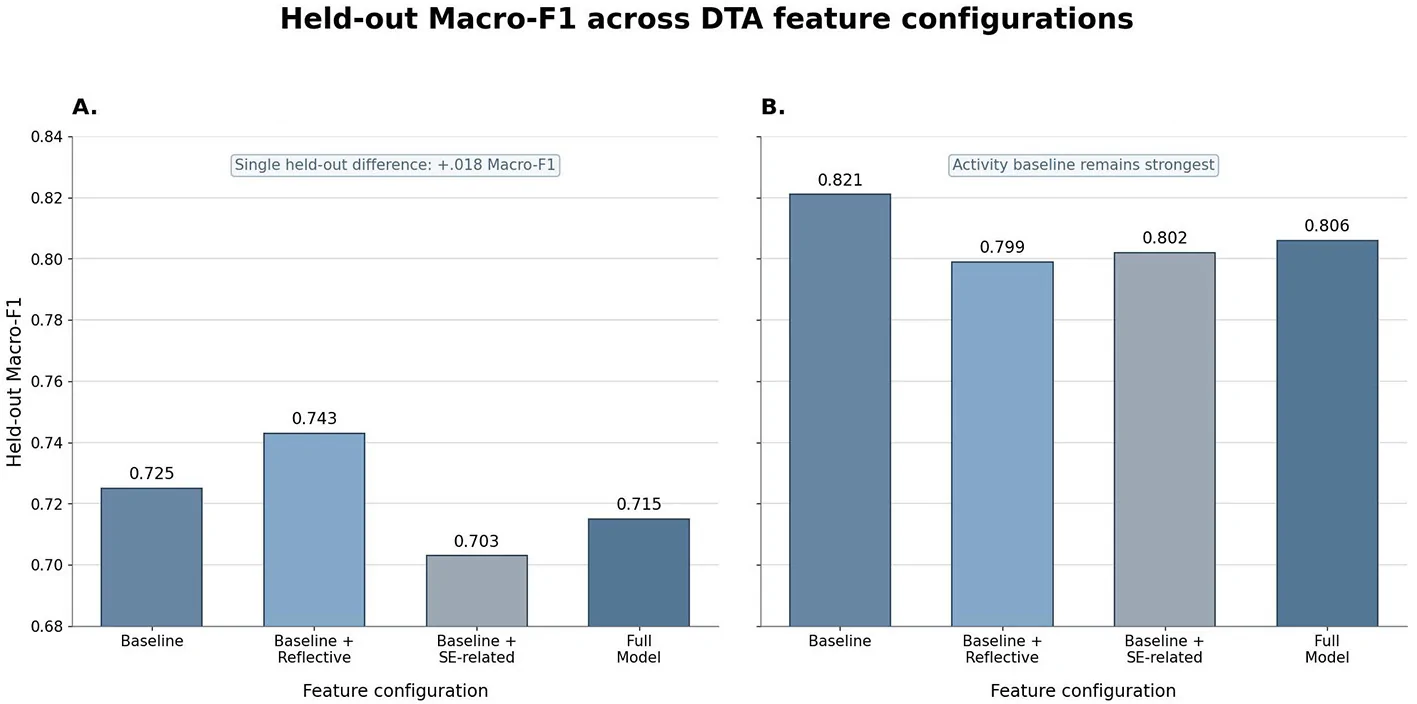
Held-out Macro-F1 across DTA feature configurations. The final-success difference is based on one held-out split; the activity-only baseline remained strongest for activity-defined engagement. **(A)** Final success prediction and **(B)** Activity-defined engagement prediction.

**Figure 4:**
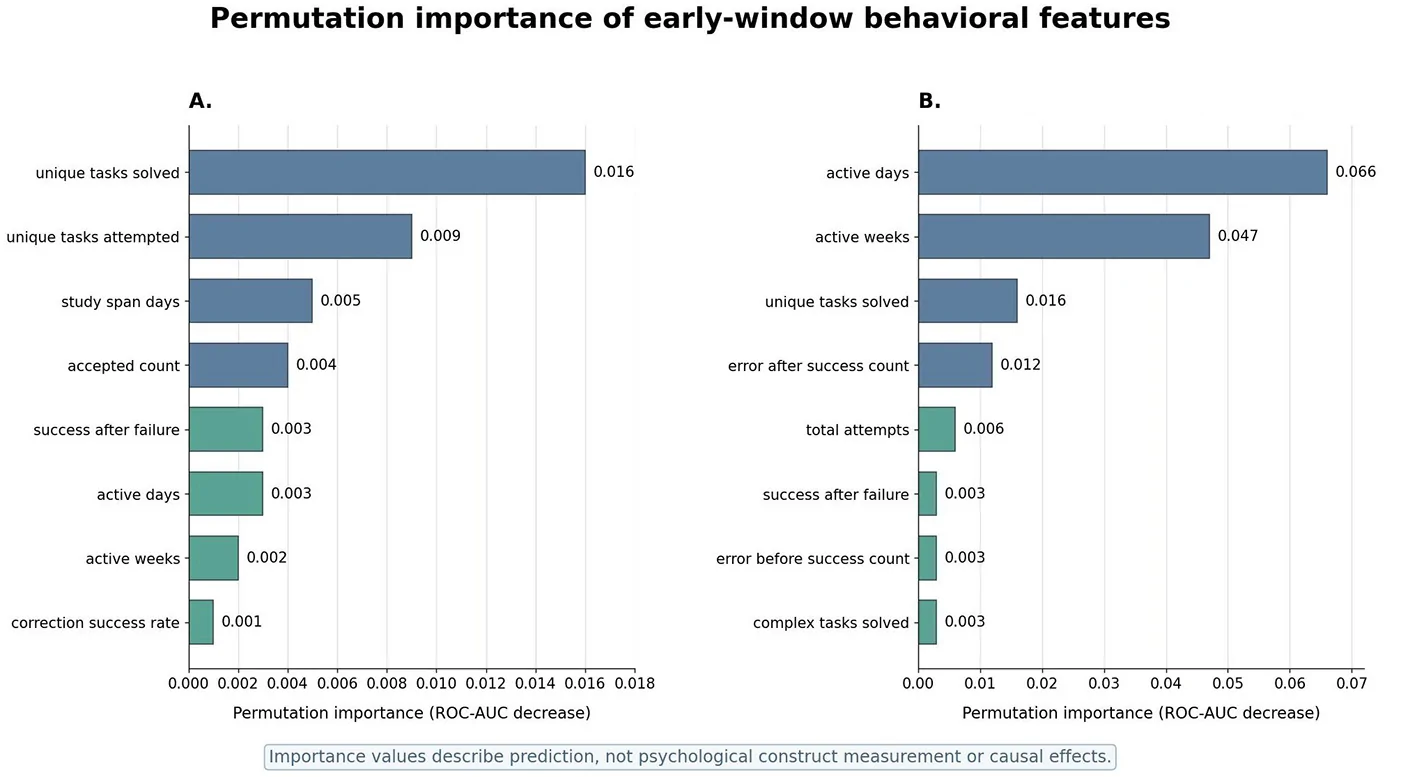
Permutation importance of early-window behavioral features. Activity-defined engagement prediction was mainly driven by activity indicators; importance values do not establish psychological measurement or causal effects. **(A)** Final success prediction and **(B)** Activity-defined engagement prediction.

The main contributions of this study are three-fold.

First, we provide an educational-psychological account of AI-supported programming that distinguishes technology acceptance from reflective AI use. Instead of treating acceptance as the final explanation of engagement, the study examines how acceptance is related to reflective feedback engagement, programming self-efficacy, and learning engagement. The survey results support strong paths from technology acceptance to reflective use and engagement, and from reflective use to programming self-efficacy, while the hypothesized indirect effects are not statistically significant. This offers a more nuanced account of AI-supported programming engagement.

Second, we use a multi-source evidence design that combines survey modeling, text-based reflective-use coding, and programming-trace prediction. The Copilot field-study analysis explores reflective use from Critical Engagement texts, while the DTA analysis models early programming behavior from log data. This design helps bridge psychological constructs and behavioral evidence, while also making clear where the evidence is strong, weak, or task-dependent.

Third, we test whether reflection-related behavioral features add predictive information for final task completion. In the DTA experiment, the held-out improvement is small and the average gain across repeated and window-sensitivity analyses is negligible. The DTA branch therefore supplies complementary programming-behavior evidence and is not a validation of reflective AI use.

The rest of this paper is organized as follows. Section 2 reviews related work on technology acceptance, programming self-efficacy, reflective learning, generative AI in programming education, and behavioral trace analytics. Section 3 presents the analytical design, including the survey model, text-based reflective-use coding, and programming-log feature construction. Section 4 describes the datasets, experimental settings, comparisons, ablation study, and further analyses. Section 5 concludes the paper and outlines future research directions.

## 2 Related work

### 2.1 Technology acceptance, self-efficacy, and engagement in AI-supported programming

Prior work on the problem addressed in this study can be grouped into four categories: technology acceptance, student engagement, programming self-efficacy, and generative AI in computing education. The first category originates from the Technology Acceptance Model, which explains system use through perceived usefulness and perceived ease of use (Davis, 1989). Later, UTAUT integrated several acceptance theories and highlighted performance expectancy, effort expectancy, social influence, and facilitating conditions as major determinants of technology use (Venkatesh et al., 2003). These models provide a strong basis for studying students' acceptance of AI-supported programming tools, but they were not designed to explain whether students use AI suggestions reflectively. The second category concerns student engagement. A widely used view treats engagement as a multidimensional construct that includes behavioral, emotional, and cognitive components (Fredricks et al., 2004), and self-regulated learning research further explains how students plan, monitor, and adjust learning strategies (Zimmerman, 2002; Pintrich, 2000; Winne and Hadwin, 1998). The third category concerns programming self-efficacy. Grounded in self-efficacy theory (Bandura, 1997), early programming research developed and validated programming-specific self-efficacy measures for novice programmers (Ramalingam and Wiedenbeck, 1998). This work shows that students' confidence in programming is educationally meaningful, although its role may change when AI tools provide immediate code suggestions. The fourth category concerns AI and LLMs in education. Broader AI-in-education reviews have described intelligent tutoring, adaptive support, and data-informed instruction as major application areas (Holmes et al., 2019; Luckin et al., 2016; Zawacki-Richter et al., 2019; Ouyang and Jiao, 2021). Recent LLM reviews further describe both the promise and risk of generative AI for learning, including personalized support, feedback generation, over-reliance, academic integrity concerns, and the need for AI literacy (Kasneci et al., 2023; Yan et al., 2024; Shi et al., 2026). High-profile discussions in Nature and Science venues have also emphasized that generative AI can reshape higher education, authorship norms, productivity, and creative work, while raising reliability and diversity concerns (Milano et al., 2023; Thorp, 2023; Noy and Zhang, 2023; Doshi and Hauser, 2024). In computing education, recent studies and reviews have examined ChatGPT, GitHub Copilot, and other code-generation tools as supports for programming tasks, while also noting concerns about foundational understanding, critical thinking, and assessment validity (Denny et al., 2024; Prather et al., 2023; Becker et al., 2023; Raihan et al., 2025; Zhu et al., 2025; Nathaniel et al., 2025). Empirical Copilot studies further show that AI coding assistants may influence productivity, behavior, and understanding, but the effects are context-dependent and often based on small or task-specific samples (Shihab et al., 2025; Aziz, 2026).

Different from studies that treat acceptance or usage as the main endpoint, the present work asks whether acceptance is connected to reflective use, programming self-efficacy, and engagement in AI-supported programming. The study also avoids assuming that all theoretical paths must be significant. Instead, it tests the psychological paths with survey data, explores text-based reflection in a Copilot field-study dataset, and examines whether related behaviors appear in programming traces. This design keeps the motivation consistent across the paper: accepting AI is not the same as engaging with AI-generated suggestions in a reflective and learning-oriented way.

### 2.2 Reflective AI use, feedback engagement, and behavioral trace evidence

The present paper is also related to three lines of work: reflective learning in programming, behavioral trace evidence, and interpretable educational prediction. First, reflection, feedback use, and self-regulation have long been regarded as important for learning complex skills (Zimmerman, 2002; Nicol and Macfarlane-Dick, 2006; Shute, 2008). Prior work on adaptive learning and self-regulated learning has shown that monitoring, feedback interpretation, and strategy adjustment are central to productive learning (Azevedo et al., 2009; Zimmerman, 2002; Pintrich, 2000). In programming education, recent work has designed tools that allow learners to replay coding processes and reflect on their strategies, errors, and improvements (Xie et al., 2023). Such studies show that reflection can make invisible problem-solving processes more visible. However, most reflection-oriented work focuses on designed interventions or qualitative learning processes, rather than linking reflective feedback engagement to AI acceptance and behavioral traces. Second, learning analytics and educational data mining have used clickstream, submission, activity, and progress features to predict student outcomes (Long and Siemens, 2011; Romero and Ventura, 2010; Baker and Yacef, 2009; Gasevic et al., 2015; Zawacki-Richter et al., 2019). In programming courses, submission logs are especially valuable because they capture attempts, errors, correction patterns, and task completion. For example, explainable prediction studies have used programming-submission features and SHAP-style explanations to interpret student performance models (Hoq et al., 2023). The DTA dataset and related work provide a large-scale source of automatically collected programming solutions and learning-process data from a (Python Software Foundation, Wilmington, DE, United States) programming course (Demidova et al., 2023). Third, explainability has become increasingly important in educational prediction, drawing from broader interpretable machine-learning work as well as educational data mining (Ribeiro et al., 2016; Lundberg and Lee, 2017; Miller, 2019; Rudin, 2019). Recent EDM and learning-analytics work emphasizes that predictive models should not only be accurate, but also interpretable and useful for instructional decision-making (Khosravi et al., 2022; Gunasekara and Saarela, 2024; Islam et al., 2025). These studies provide important methodological foundations, but many still rely mainly on generic activity indicators, and fewer explicitly test whether theoretically grounded reflective behaviors add value beyond basic engagement features.

The present work differs from this literature by using behavioral trace evidence as a cautious complement to psychological analysis rather than as a replacement or validation of psychological measurement. It constructs baseline activity, recovery-related, and persistence-related feature groups and tests their incremental prediction through controlled ablation. The feature names are theory-informed descriptions of log patterns, not measurements of latent reflective AI use or self-efficacy.

## 3 Methods

### 3.1 Overview of the analytical design

We organize the study through a Reflective AI Acceptance Integration Framework, abbreviated as RAIAF. RAIAF is an evidence-integration design rather than a new machine-learning architecture or a claim that the three datasets measure the same process. The datasets contain different participants and contexts and are not pooled. The survey and Copilot modules concern AI-supported programming, whereas the DTA module contains programming behavior without a direct measure of AI use.

RAIAF consists of three modules. The first module estimates the relationships among technology acceptance (TA), reflective use (RU), programming self-efficacy (PSE), and engagement (ENG). The second module explores reflective use through open-text responses in the Copilot field-study dataset. The third module transforms DTA programming logs into early-window behavioral trace features and predicts later learning outcomes as complementary evidence about recovery and persistence in programming, not as validation or replication of reflective AI use.

Figure 5 summarizes the analytical design. The figure separates survey modeling, text-based reflection coding, and log-based behavioral trace prediction while keeping the same theoretical motivation across all three branches.

**Figure 5:**
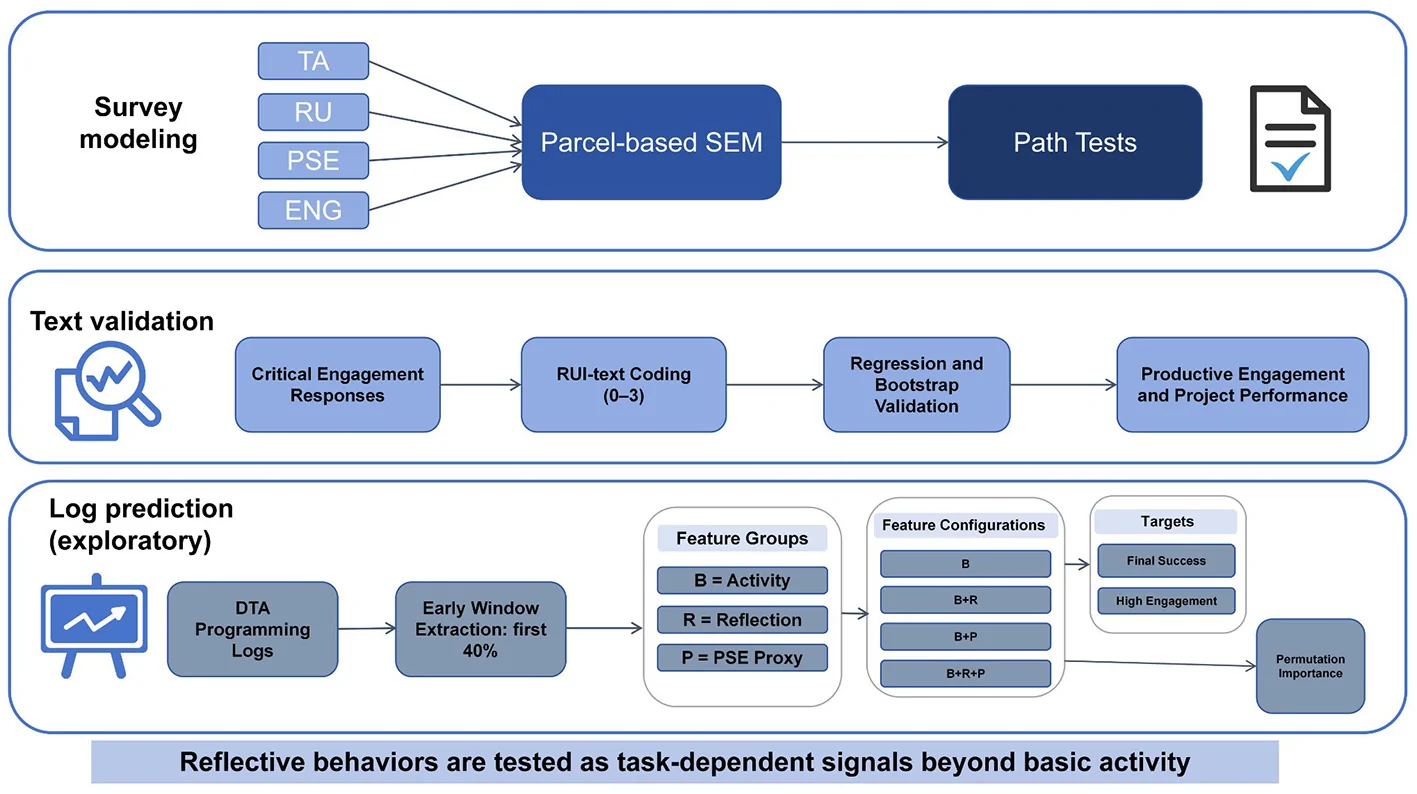
Analytical design of RAIAF. The survey branch tests the relationships among technology acceptance (TA), reflective use (RU), programming self-efficacy (PSE), and engagement (ENG) using parcel-based SEM. The text branch codes Critical Engagement responses into RUI-text scores for exploratory evidence. The log branch extracts early-window activity, recovery, and persistence indicators from DTA logs to predict final success and activity-defined engagement. B, baseline activity features; R, recovery-related behavioral features; P, persistence-related indicators.

Let *i* index students, *j* index survey items, *t* index time-stamped programming events, and *y*_*i*_ denote a prediction target. The four main psychological constructs are represented as:


$$\begin{array}{l} \eta_{i}=\left\{TA_{i},RU_{i},PSE_{i},ENG_{i}\right\}. \end{array}$$

For item-level survey responses, each item score *x*_*ij*_ is standardized as:


$$\begin{array}{l} z_{ij}=\frac{x_{ij}-\mu_{j}}{\sigma_{j}}, \end{array}$$

where μ_*j*_ and σ_*j*_ are the item mean and standard deviation.

The observed composite score of construct *c* for student *i* is calculated as:


$$\begin{array}{l} S_{ic}=\frac{1}{\left|I_{c}\right|}\underset{j\in I_{c}}{\sum }z_{ij}, \end{array}$$

where $I_{c}$ is the item set for construct *c*. This construction was used for descriptive analysis and path-based robustness checks.

The main latent model used *domain-representative parcels* defined before model comparison: perceived usefulness (PU) and perceived ease of use (PEU) served as the two TA indicators, and cognitive (CE), behavioral (BE), and emotional engagement (EE) served as the three ENG indicators. RU and PSE remained item-level latent variables. This decision preserved the established subdimensions of TA and engagement and reduced the number of freely estimated item-level parameters in the fixed secondary sample (Little et al., 2002; Matsunaga, 2008). Parceling was not used as evidence that the item-level model fit well. The item-level model and its weak fit are reported explicitly, and substantive conclusions are conditioned on that measurement limitation.

The measurement model can be written as:


$$x_{i}=\Lambda \eta_{i}+\epsilon_{i}$$

where **x**_*i*_ denotes observed indicators or parcels, Λ is the factor-loading matrix, and ϵ_*i*_ is measurement error.

The structural component of the survey model is specified as:


$$\begin{array}{lll} RU_{i} & = & \alpha_{1}+a_{1}TA_{i}+\varepsilon_{1i},\\ PSE_{i} & = & \alpha_{2}+a_{2}TA_{i}+d_{21}RU_{i}+\varepsilon_{2i},\\ ENG_{i} & = & \alpha_{3}+c^{\prime }TA_{i}+b_{1}RU_{i}+b_{2}PSE_{i}+\varepsilon_{3i}. \end{array}$$

The total indirect effect from technology acceptance to engagement is decomposed into three paths:


$$\begin{array}{l} IE_{total}=a_{1}b_{1}+a_{2}b_{2}+a_{1}d_{21}b_{2}. \end{array}$$

$$\begin{array}{l} TE=c^{\prime }+IE_{total}. \end{array}$$

This formulation allows us to test whether technology acceptance is linked to engagement directly, indirectly through reflective use, indirectly through programming self-efficacy, or through a serial process involving both constructs.

### 3.2 Text-based reflective use

The Copilot field-study text module uses open-ended Critical Engagement responses to build a text-based reflective-use score. The purpose is not to replace the survey construct, but to explore whether reflective use can also be observed from students' written responses to AI suggestions.

Each response is coded into a four-level reflective-use index:


$$\begin{array}{l} RUI_{i}^{text}\in \left\{0,1,2,3\right\}, \end{array}$$

where 0 indicates that a nonblank response contains no observable reflective evidence, 1 indicates recognition of an AI suggestion or issue, 2 indicates explanation or correction, and 3 indicates comparison, revision, or transferable understanding. Blank Critical Engagement responses are treated as missing and are excluded from coding, reliability estimation, regression, and bootstrap analysis; they are not assigned a reflection score.

The English responses were presented to the coders in Chinese translation, with programming tokens and code examples preserved. Two human coders independently rated all 66 nonblank responses using a manual that defined category boundaries, typical evidence, and exclusion rules. Both coders were blinded to the other coder's scores. Because the four categories are ordered, initial intercoder reliability was evaluated using linear-weighted Cohen's kappa, supplemented by exact agreement and Krippendorff's ordinal alpha. Discrepant cases were adjudicated after the initial reliability calculation, and the consensus scores were used in the text regressions. The 48 blank responses remained missing throughout the inferential analysis.

The text-analysis regression is specified as:


$$\begin{array}{lll} RUI_{i}^{text} & = & \gamma_{0}+\gamma_{1}TA_{i}+u_{i},\\ PE_{i} & = & \delta_{0}+\delta_{1}TA_{i}+\delta_{2}RUI_{i}^{text}+\delta_{3}LS_{i}+v_{i}, \end{array}$$

where *PE*_*i*_ denotes productive engagement and *LS*_*i*_ denotes perceived learning support. The text-based indirect effect is:


$$\begin{array}{l} IE^{text}=\gamma_{1}\delta_{2}. \end{array}$$

### 3.3 Log-based behavioral trace analysis

The DTA behavioral trace module models student behavior from time-stamped programming logs. Because DTA neither measures AI use nor contains the participants from the survey or Copilot datasets, this branch is interpreted only as complementary evidence about programming behavior. Features are extracted using a common course-calendar cutoff rather than each student's eventual last timestamp. Let *t*_0_ and *T*_*C*_ be the first and last timestamps in the course-wide log and let ρ be the calendar-window ratio. The primary analysis used ρ = 0.40.

The early learning window is defined as:


$$\begin{array}{l} W_{i}\left(\rho \right)=\left\{e_{it}:t\leq t_{0}+\rho \left(T_{C}-t_{0}\right)\right\}, \end{array}$$

where *e*_*it*_ is a programming event for student *i*. The cutoff depends only on the published course calendar represented by the complete log and not on an individual student's future final activity time. Sensitivity analyses repeat the procedure at ρ = 0.30 and ρ = 0.50.

RAIAF constructs three feature groups. The baseline feature group contains activity and progress indicators:


$$x_{i}^{B}=\left[attempts_{i},\;accepted_{i},\;errorBeforeSuccess_{i},\;errorAfterSuccess_{i},tasksAttempted_{i},\;activeDays_{i},\;activeWeeks_{i},\;tasksSolved_{i},studySpan_{i},\;regularity_{i}\right]$$

The reflection-related feature group captures recovery and revision patterns after unsuccessful attempts:


xiR=[retryAfterFailurei, successAfterFailurei, retry48hi,medianRetryIntervali, correctionSuccessRatei,persistenceAfterFailurei, RUIbehaviori]

The self-efficacy-related behavioral indicator group contains variables that may approximate confidence-related persistence and recovery:


$$x_{i}^{P}=\left[complexTasksSolved_{i},\;correctionSuccessRate_{i},persistenceAfterFailure_{i},\;PSEproxy_{i}\right]$$

Thus, the Baseline, Baseline + Reflective, and Baseline + SE-related configurations contain 10, 17, and 14 features, respectively. Because correction success rate and persistence after failure occur in both theoretically motivated augmented groups, the union in the Full Model contains 19 unique features rather than 21. These variables are behavioral approximations only and are not direct psychological measurements of reflective AI use or programming self-efficacy.

The full feature representation is formed by feature-level fusion:


$$x_{i}^{Full}=\left[x_{i}^{B}⊕x_{i}^{R}⊕x_{i}^{P}\right]$$

where ⊕ denotes concatenation.

For the activity-defined engagement label, the full-period activity score is computed as the mean of standardized activity indicators:


$$E_{i}=\frac{1}{5}\left(z\left(totalAttempts_{i}\right)+z\left(activeDays_{i}\right)+z\left(activeWeeks_{i}\right)+z\left(uniqueTasksAttempted_{i}\right)+z\left(regularity_{i}\right)\right)$$

Students at or above the 67th percentile are labeled as activity-defined engagement:


$$\begin{array}{l} y_{i}^{ADE}=I\left(E_{i}\geq Q_{0.67}\left(E\right)\right). \end{array}$$

This target is not treated as an independent psychological criterion because its definition overlaps with the activity variables used by the Baseline model. It is retained as a positive activity benchmark and as a boundary test of whether recovery-related features add information beyond overlapping activity indicators.

Final success is defined as completing all 11 programming tasks:


$$\begin{array}{l} y_{i}^{FS}=I\left(tasksSolved_{i}=11\right). \end{array}$$

For binary prediction, the model estimates:


$${\hat{p}}_{i}=P\left(y_{i}=1|x_{i}\right)=f_{\theta }\left(x_{i}\right),$$

where *f*_θ_ is a classifier selected from conventional behavioral trace models. The empirical objective is:


$$\begin{array}{l} L\left(\theta \right)=-\frac{1}{N}\overset{N}{\underset{i=1}{\sum }}\left[y_{i}\log{\hat{p}}_{i}+\left(1-y_{i}\right)\log\left(1-{\hat{p}}_{i}\right)\right]+\lambda \Omega \left(\theta \right). \end{array}$$

The Brier score is used to assess calibration:


$$\begin{array}{l} Brier=\frac{1}{N}\overset{N}{\underset{i=1}{\sum }}{\left({\hat{p}}_{i}-y_{i}\right)}^{2}. \end{array}$$

Permutation importance for feature *k* is defined as the decrease in predictive performance after randomly permuting that feature:


$$PI_{k}=M\left(X,y\right)-M\left(\pi_{k}\left(X\right),y\right),$$

where *M* is the evaluation metric and π_*k*_(**X**) denotes the feature matrix after permuting feature *k*.

### 3.4 Algorithmic procedure

The complete RAIAF analytical workflow is summarized in Algorithm 1.

Algorithm 1 RAIAF analytical workflow. 
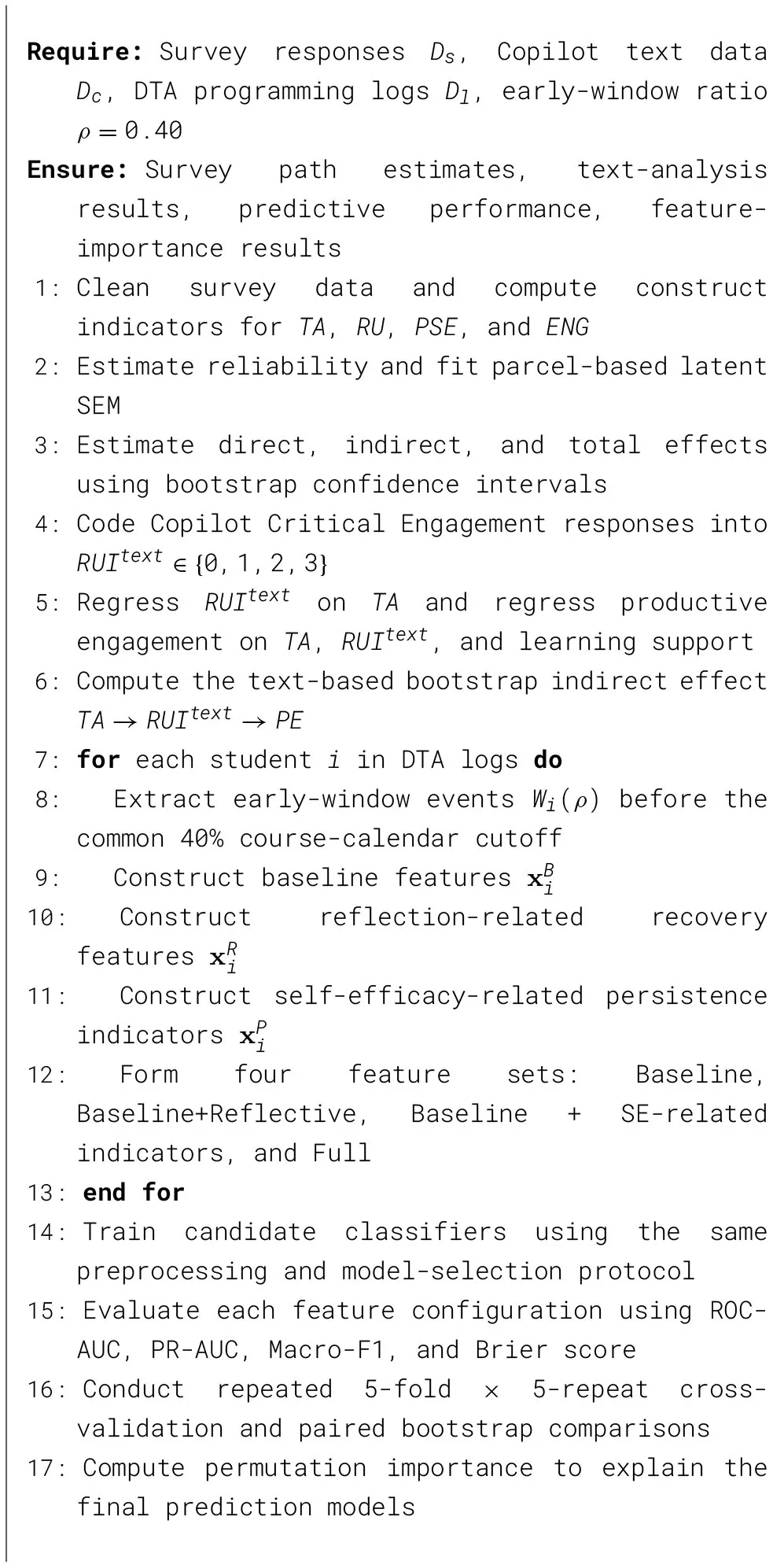

### 3.5 Methodological rationale

The rationale of RAIAF is three-fold. First, survey modeling tests whether the proposed psychological mechanism is supported at the construct level. Second, text-based coding checks whether reflective use can be observed beyond closed-ended survey items. Third, log-based prediction asks whether recovery-related programming behaviors provide incremental predictive information. Because DTA does not measure AI use, the third module cannot confirm the first two.

The method is also conservative. It does not claim that all indirect effects are significant. It reports when a hypothesized path is not supported. It also treats self-efficacy-related behavioral indicators as approximations, not as direct replacements for psychological self-efficacy scales. This distinction is important for maintaining construct validity.

## 4 Experiments

### 4.1 Experimental overview

The experiments were designed to answer three questions. First, does the survey-based psychological model support the proposed links among technology acceptance, reflective use, programming self-efficacy, and engagement? Second, can text-based reflective use in the Copilot field-study data provide exploratory evidence of reflection in AI-supported programming? Third, do recovery and persistence indicators add early predictive information in DTA programming logs?

The experiments combine latent-variable modeling, exploratory text coding, and complementary behavioral trace prediction. Only the first two modules directly address AI-supported programming.

### 4.2 Datasets

#### 4.2.1 Dataset A: AI-supported programming survey

Dataset A was an AI-supported programming learning survey dataset from Mendeley Data (Tang et al., 2026). It contained *N* = 131 valid responses. Four constructs were used in the main analysis: technology acceptance (TA), reflective use (RU), programming self-efficacy (PSE), and learning engagement (ENG). TA was measured with six items, RU with three items, PSE with four items, and ENG with nine items.

The internal consistency of the item sets was high. Cronbach's alpha was 0.938 for TA, 0.951 for RU, 0.962 for PSE, and 0.957 for ENG. The parcel-based latent SEM had CFI = 0.951, TLI = 0.934, RMSEA = 0.082, and SRMR = 0.029. The model-fit results are summarized in Table 1. However, the item-level MLR model showed weak incremental fit (CFI = 0.889; TLI = 0.874), so parcel-model results are not presented as resolving all measurement-model concerns. An item-level WLSMV sensitivity model for the ordered responses produced CFI = 0.996, TLI = 0.995, RMSEA = 0.067, and SRMR = 0.039, but it yielded negative observed-variable variances and a non-positive-definite parameter covariance matrix; this inadmissible solution was not used for inference.

**Table 1:** Survey model-fit summary.

Model	*N*	χ^2^	*df*	CFI	TLI	RMSEA	SRMR
Item four-factor SEM	131	391.102	203	0.889	0.874	0.084	0.045
**Parcel latent SEM**	**131**	**92.598**	**49**	**0.951**	**0.934**	**0.082**	**0.029**
Parcel scale SEM	131	13.035	11	0.994	0.989	0.038	0.019

The parcel model preserves predefined subdimensions, but the weak item-level MLR fit remains a measurement limitation. Bold values indicate the primary parcel-based latent SEM used for inference.

All 22 survey items had complete data; observed response ranges contained three to five of the five Likert categories. In the parcel model, standardized loadings ranged from 0.884 to 0.956 (Table 2). HTMT values were below 0.90 for every construct pair (Henseler et al., 2015). The full HTMT discriminant-validity matrix is reported in Table 3. The TA–ENG value was 0.870, which exceeds the more conservative 0.85 guideline and indicates only borderline separation under that stricter criterion.

**Table 2:** Standardized loadings in the parcel-based latent SEM.

Construct	Indicator	Loading
TA	PU	0.911
TA	PEU	0.893
RU	RU1	0.934
RU	RU2	0.908
RU	RU3	0.956
PSE	PSE1	0.935
PSE	PSE2	0.930
PSE	PSE3	0.930
PSE	PSE4	0.930
ENG	CE	0.934
ENG	BE	0.884
ENG	EE	0.949

**Table 3:** HTMT discriminant-validity ratios.

Construct	TA	RU	PSE	ENG
TA	1.000			
RU	0.751	1.000		
PSE	0.680	0.755	1.000	
ENG	0.870	0.826	0.704	1.000

#### 4.2.2 Dataset B: copilot field-study text evidence

Dataset B was the GitHub Copilot field-study dataset from Mendeley Data (Aziz, 2026). The student-level file contained *N* = 114 records, of which 66 contained nonblank Critical Engagement text and 48 were blank. Two human coders independently coded the 66 nonblank responses into *RUI*^*text*^ scores ranging from 0 to 3. Before adjudication, the coders agreed exactly on 38 of 66 responses (57.6%); 95.5% of ratings differed by no more than one category. Linear-weighted Cohen's κ was 0.421, and Krippendorff's ordinal α was 0.495. The 28 disagreements were resolved by consensus after reliability was calculated. The 48 blank responses remained missing and were excluded from text inference. Among the 66 coded texts, the consensus distribution was 8 cases coded as 0, 38 as 1, 16 as 2, and 4 as 3.

The mean technology acceptance score in Dataset B was 4.730, with a standard deviation of 0.255 on a five-point scale. Of the 114 students, 33 (28.9%) had the maximum score of 5 and 93 (81.6%) scored at least 4.5. This concentration at the upper end constitutes a ceiling effect and sharply restricts between-student variance. The mean *RUI*^*text*^ score among the 66 nonblank responses was 1.242.

The reliability estimates describe the two coders' independent ratings before discussion. Consensus scores were used only to form the final analysis variable and were not used to recalculate or inflate intercoder reliability.

#### 4.2.3 Dataset C: DTA programming behavioral traces

Dataset C was the DTA programming-log dataset, a public programming-submission dataset generated by a Digital Teaching Assistant environment (Demidova et al., 2023). It does not contain a measure of AI-assistant use. The full student-level sample contained *N* = 1, 423 students, and the 40% course-calendar window contained early activity for *N* = 1, 122 students. Features were extracted before one common cutoff defined from the course-wide log range.

Two prediction targets were used. The first target, *final*_*success*_*all*_11, indicated whether a student solved all 11 programming tasks. The second target, renamed *activity*_*defined*_*engagement*, was constructed from the full-period activity score. This score was the mean of standardized total attempts, active days, active weeks, unique tasks attempted, and regularity. Students at or above the 67th percentile were assigned the activity-defined outcome. Because the label derives from activity intensity and regularity, it is not interpreted as the broader psychological construct of engagement.

Table 4 summarizes the role of each dataset in the empirical design. The table also clarifies why the evidence should be interpreted as complementary rather than as a single pooled model.

**Table 4:** Datasets and analysis roles in RAIAF.

Dataset	Sample	Main evidence	Analysis role
Survey SEM	*N* = 131	TA, RU, PSE, and ENG scale indicators	Tests the psychological path model and indirect-effect assumptions.
Copilot field study	*N* = 114; 66 texts	Critical engagement responses and project outcomes	Provides exploratory text-based evidence of reflective use.
DTA logs	*N* = 1, 423; early-window *N* = 1, 122	Time-stamped submissions, task states, and early behavior features	Evaluates predictive value and explainability of behavior features.

### 4.3 Evaluation metrics

For the survey model, we report Cronbach's alpha, model fit indices, standardized path estimates, and bootstrap confidence intervals for indirect effects. Cronbach's alpha is used as an internal-consistency index:


$$\begin{array}{c} \alpha =\frac{K}{K-1}\left(1-\frac{\overset{K}{\underset{j=1}{\sum }}\sigma_{j}^{2}}{\sigma_{total}^{2}}\right), \end{array}$$

where *K* is the number of items, $\sigma_{j}^{2}$ is item variance, and $\sigma_{total}^{2}$ is total score variance.

For the behavioral trace prediction models, four metrics are reported. ROC-AUC measures the ranking quality between positive and negative cases. PR-AUC is useful when positive cases are not evenly distributed. Macro-F1 gives equal weight to each class:


$$\begin{array}{c} Macro\text{-}F1=\frac{1}{C}\overset{C}{\underset{c=1}{\sum }}\frac{2Precision_{c}Recall_{c}}{Precision_{c}+Recall_{c}}. \end{array}$$

The Brier score measures probability calibration and is lower when predictions are better calibrated.

### 4.4 Experimental settings

The survey analysis was conducted in R 4.6.1 with lavaan 0.7–2. The main parcel-based latent SEM was estimated with robust maximum likelihood (MLR), treating the five-point Likert responses and their predefined parcel means as approximately continuous (Rhemtulla et al., 2012). Direct paths are reported with MLR robust standard errors, two-sided *p* values, and 95% robust Wald confidence intervals. Indirect and total effects were re-estimated with maximum likelihood and 5,000 case-resampling bootstrap draws; percentile intervals, rather than normal-approximation bootstrap *p* values, determine indirect-effect significance. All 22 modeled item responses were complete, so the specified full-information maximum-likelihood missing-data option did not change the effective sample.

No a priori power analysis was possible because the public secondary dataset fixed the sample at *N* = 131. SEM sample requirements depend on loading strength, model complexity, missingness, and effect size rather than a universal ratio (Wolf et al., 2013). With four constructs and a three-coefficient serial indirect effect, this sample was considered adequate only for exploratory estimation of large component paths and likely underpowered for small mediation effects (Fritz and MacKinnon, 2007). The lower-complexity parcel model was therefore paired with transparent item-level and scale-score sensitivity models; it was not used to claim that *N* = 131 validates the full measurement structure.

The Copilot text analysis was exploratory. Consensus-based coding formed a four-level *RUI*^*text*^ score for the 66 nonblank responses; the 48 blank responses remained missing. Complete-case regressions tested whether technology acceptance predicted *RUI*^*text*^ and whether *RUI*^*text*^ predicted productive engagement and project score. The text-based indirect effect used 5,000 case-resampling bootstrap draws and percentile confidence intervals.

For DTA prediction, labels were constructed from the full course period, whereas predictors were restricted to the common early calendar window. A stratified 70% development/30% held-out split was created once with seed 20260523. Median imputation and standardization were implemented inside each scikit-learn pipeline and fitted only on the relevant training fold. The held-out observations were never used for preprocessing, learner selection, hyperparameter selection, or feature-configuration selection.

Within the 70% development set, five-fold stratified grid search compared elastic-net logistic regression, random forest, and histogram-based gradient boosting using ROC-AUC. The logistic grid used *C*∈{0.1, 1, 3} and *l*1_*ratio*∈{0, 0.5, 1}; random forest used *n*_*estimators*∈{200, 500}, *max*_*depth*∈{3, 5}, and *min*_*samples*_*leaf*∈{2, 5}; histogram-based gradient boosting used *learning*_*rate*∈{0.03, 0.1}, *max*_*iter*∈{100, 200}, and *max*_*leaf*_*nodes*∈{15, 31}. The selected pipeline was refitted on the complete development set and evaluated once on the untouched test set. A repeated five-fold analysis with the locked learner and hyperparameters was used only as a descriptive stability analysis, not as a second independent test. The 30%, 40%, and 50% calendar-window sensitivity analysis used the same locked specifications. Python analyses used scikit-learn with seed 20260523 in a CPU environment.

### 4.5 Analytical comparisons

#### 4.5.1 Comparison with existing methods

The comparison was organized around theoretically interpretable baseline models. At the survey level, the technology-acceptance-only path served as a baseline explanation of engagement. At the behavioral level, the activity-only trace model served as the main baseline, and augmented configurations added recovery and persistence indicators.

Because no established public benchmark defines the same survey constructs, Copilot text labels, and DTA outcome targets together, we used two complementary comparison strategies. First, the survey and text models were compared against theoretically simpler paths, such as technology-acceptance-only and activity-only explanations. Second, the DTA experiment compared 24 learner-feature configurations formed by three candidate learners and four feature configurations across two prediction targets. Table 5 reports the cross-validated ROC-AUC values used during model selection.

**Table 5:** Candidate learner comparison for DTA model selection.

Feature configuration	No. of features	Final success CV ROC-AUC			Activity-defined engagement CV ROC-AUC		
		ElasticNet	Random forest	HGB	ElasticNet	Random forest	HGB
Baseline	10	0.794	**0.799**	0.776	0.871	0.866	0.854
Baseline + reflective	17	0.786	0.796	0.769	**0.872**	0.864	0.856
Baseline + SE-related indicators	14	0.798	0.795	0.771	0.869	0.866	0.850
Full model	19	0.790	0.795	0.769	0.870	0.863	0.856

Bold values indicate the strongest cross-validated ROC-AUC within each target.

Because the three public datasets do not share a single published benchmark, we used a comparison strategy based on theoretically simpler alternatives rather than claiming a universal leaderboard. Table 6 summarizes representative comparison settings across the survey, text, and DTA modules. The comparison covers model-fit variants, text-analysis paths, and conventional behavioral trace classifiers, including regularized linear models, random forests, and histogram-based gradient boosting. This provides more than ten comparison settings across multiple datasets while keeping the analysis aligned with the theoretical purpose of RAIAF.

**Table 6:** Comparison settings across the RAIAF modules.

Dataset/module	Comparison setting	Metric	Value	Role
Survey	Item four-factor SEM	CFI	0.889	Item-level baseline
Survey	**Parcel latent SEM**	CFI	**0.951**	Recommended SEM
Survey	Parcel scale SEM	CFI	0.994	Low-complexity robustness check
Copilot text	TA → *RUI*^*text*^	Coef.	0.690	Text antecedent test (*N* = 66)
Copilot text	*RUI*^*text*^ → productive engagement	Coef.	0.149	Text-analysis path (*N* = 66)
Copilot text	TA → *RUI*^*text*^ → productive engagement	Indirect estimate	0.101	Bootstrap text mediation
DTA final success	Logistic ElasticNet + Baseline	CV ROC-AUC	0.794	Linear baseline
DTA final success	**Random Forest** **+** **Baseline**	CV ROC-AUC	**0.799**	Tree baseline
DTA final success	Hist. Gradient Boosting + Baseline	CV ROC-AUC	0.776	Boosting baseline
DTA final success	Random Forest + Reflective	CV ROC-AUC	0.796	Reflective variant
DTA final success	Logistic ElasticNet + SE-related indicators	CV ROC-AUC	0.798	Persistence-indicator variant
DTA activity-defined engagement	Logistic ElasticNet + Baseline	CV ROC-AUC	0.871	Activity baseline
DTA activity-defined engagement	**Logistic ElasticNet** **+** **Reflective**	CV ROC-AUC	**0.872**	Recovery-feature variant
DTA activity-defined engagement	Logistic ElasticNet + Full	CV ROC-AUC	0.870	Full feature variant

Higher values are better for CFI and CV ROC-AUC. For text analysis, the table reports the main coefficient or indirect-effect estimate because no standard benchmark metric exists. Bold values indicate the primary or recommended setting selected for interpretation within each module or prediction target.

Table 7 reports the MLR direct paths. Technology acceptance predicted reflective use (β = 0.774, *p* < 0.001) and engagement (β = 0.694, *p* < 0.001). Reflective use predicted programming self-efficacy (β = 0.519, *p* < 0.001) and engagement (β = 0.273, *p* = 0.034). The direct TA → PSE path was not significant (*p* = 0.057), and PSE did not predict engagement (*p* = 0.813). These direct associations do not establish mediation.

**Table 7:** Survey SEM direct paths estimated with MLR.

Path	Std. β	Robust SE	*p*	Wald LLCI	Wald ULCI
TA → RU	**0.774**	0.111	< 0.001	0.569	1.003
TA → PSE	0.309	0.263	0.057	–0.016	1.016
RU → PSE	**0.519**	0.201	< 0.001	0.432	1.221
TA → ENG	**0.694**	0.178	< 0.001	0.364	1.061
RU → ENG	**0.273**	0.131	0.034	0.021	0.532
PSE → ENG	0.021	0.056	0.813	–0.097	0.124

Confidence intervals and standard errors are robust and refer to unstandardized estimates; β is standardized. Bold values indicate statistically significant standardized path coefficients at *p* < 0.05.

Table 8 shows the indirect-effect tests. The total effect of TA on ENG was significant, but the indirect effects through RU, through PSE, and through the serial RU–PSE pathway were not statistically significant. Therefore, the results should be described as partial support for the framework, not as evidence of significant mediation.

**Table 8:** Indirect effects in the survey model.

Effect path	Estimate	Boot SE	Boot LLCI	Boot ULCI
TA → RU → ENG	0.217	0.162	–0.065	0.436
TA → PSE → ENG	0.007	0.081	–0.164	0.060
TA → RU → PSE → ENG	0.009	0.041	–0.082	0.075
Total indirect effect	0.232	0.208	–0.233	0.447
Total effect	**0.945**	0.098	0.791	1.174

Percentile confidence intervals are based on 5,000 bootstrap resamples; an interval spanning zero is interpreted as non-significant. Bold values indicate effects whose 95% bootstrap confidence interval does not include zero.

Figure 2 visualizes the survey-based structural path model. It highlights the significant direct paths and clarifies that the hypothesized indirect effects through RU, PSE, and their serial pathway were not statistically significant.

Table 9 reports the Copilot analysis of the 66 nonblank texts. Technology acceptance did not significantly predict *RUI*^*text*^ (*b* = 0.690, *SE* = 0.435, and *p* = 0.118), and *RUI*^*text*^ did not significantly predict productive engagement or project score. The indirect estimate was 0.101, with a 5,000-resample percentile interval from –0.022 to 0.304. Thus, after treating blank responses as missing, the text analysis does not provide evidence for either the antecedent path or a text-based mediation pathway.

**Table 9:** Copilot field-study regression and indirect-effect results.

Outcome	Predictor	Coef.	SE	*p*	*N*
*RUI* ^*text*^	Technology acceptance	0.690	0.435	0.118	66
Productive engagement	Technology acceptance	0.110	0.479	0.818	66
Productive engagement	*RUI* ^*text*^	0.149	0.116	0.202	66
Project score	*RUI* ^*text*^	0.047	0.073	0.524	66
Project score	Completion time	0.082	0.050	0.108	66
Indirect effect	TA → *RUI*^*text*^ → productive engagement	0.101	–	[–0.022, 0.304]	66

#### 4.5.2 Behavioral trace baselines

In the DTA experiment, the activity-only Baseline represents a strong behavioral trace baseline. It uses early activity and progress indicators without reflection-related recovery features or self-efficacy-related persistence indicators. The proposed variants add theoretically motivated features to this baseline.

Table 10 shows the single untouched-test results. For *final*_*success*_*all*_11, Baseline + Reflective increased Macro-F1 from 0.725 to 0.743 and changed the Brier score from 0.177 to 0.175. These absolute differences are small and are not evidence of educationally important improvement. The Full Model achieved the highest ROC-AUC (0.808), but it did not outperform all other configurations across all metrics. For *activity*_*defined*_*engagement*, the Baseline model was strongest across the held-out metrics, as expected from the overlap between its predictors and the outcome definition.

**Table 10:** Held-out comparison on DTA behavioral trace targets.

Target	Model	ROC-AUC	PR-AUC	Macro-F1	Brier
*final*_*success*_*all*_11	Baseline	0.803	0.926	0.725	0.177
	Baseline + Reflective	0.807	0.927	**0.743**	**0.175**
	Baseline + SE-related indicators	0.803	**0.929**	0.703	0.182
	Full Model	**0.808**	0.928	0.715	0.176
*activity*_*defined*_*engagement*	Baseline	**0.911**	**0.893**	**0.821**	**0.118**
	Baseline + Reflective	0.908	0.887	0.799	0.121
	Baseline + SE-related indicators	0.908	0.887	0.802	0.121
	Full Model	0.910	0.889	0.806	0.120

Bold values indicate the best held-out performance within each target and metric; lower values are better for the Brier score.

Figure 3 gives a visual comparison of held-out Macro-F1 values across feature configurations. It displays the small single-test difference for final success and the stronger activity-only baseline for activity-defined engagement.

### 4.6 Ablation study

The ablation study evaluates the contribution of each feature group. The Baseline model uses activity and progress features. Baseline + Reflective is retained as the configuration name in result files but adds recovery-related variables; it is not a measured reflection construct. Baseline + SE-related adds behavioral persistence approximations, and the Full Model combines the unique variables.

For final-success prediction, Baseline + Reflective showed a 0.018 held-out Macro-F1 difference. The original repeated analysis showed a mean difference of 0.006, whereas the calendar-window rerun under the current locked pipeline showed a 40% difference of 0.001. This sensitivity to the evaluation run, together with the very small Brier differences, indicates statistical rather than practical importance. No intervention benefit or educationally meaningful gain is inferred.

The SE-related behavioral indicator group provided weaker evidence. For final success, it had similar ROC-AUC and slightly higher PR-AUC than Baseline in the held-out split, but Macro-F1 and Brier score were worse. These mixed differences do not support a stable advantage.

For activity-defined engagement prediction, none of the augmented feature sets materially improved the activity-only Baseline. Repeated cross-validation produced almost identical ROC-AUC values across configurations. The complete repeated cross-validation results are reported in Table 11. This pattern reflects the label definition: baseline features already represent the same activity domain. The target is retained only as a positive activity benchmark and boundary test, not as an independent test of psychological engagement.

**Table 11:** Repeated cross-validation results for DTA prediction.

Target	Model	ROC-AUC	PR-AUC	Macro-F1	Brier
*final*_*success*_*all*_11	Baseline	0.799 ± 0.032	**0.924**±0.014	0.696 ± 0.027	0.176 ± 0.012
	Baseline + Reflective	0.798 ± 0.032	0.923 ± 0.014	**0.702**±0.028	**0.175**±0.012
	Baseline + SE-related indicators	**0.801**±0.031	0.922 ± 0.014	0.684 ± 0.029	0.180 ± 0.012
	Full Model	0.798 ± 0.033	0.923 ± 0.015	0.700 ± 0.030	**0.175**±0.012
*activity*_*defined*_*engagement*	Baseline	**0.882**±0.025	**0.857**±0.032	0.783 ± 0.034	**0.136**±0.016
	Baseline + Reflective	0.882 ± 0.026	0.856 ± 0.032	0.782 ± 0.034	0.136 ± 0.015
	Baseline + SE-related indicators	0.882 ± 0.026	0.854 ± 0.033	0.783 ± 0.032	0.137 ± 0.015
	Full Model	0.882 ± 0.026	0.855 ± 0.033	**0.785**±0.032	**0.136**±0.015

Bold values indicate the strongest mean repeated-cross-validation performance within each target and metric; lower values are better for the Brier score.

The ablation results therefore support only a cautious, task-dependent interpretation. Recovery-related variables may contain some incremental signal for final completion, but their average contribution is small and unstable across evaluation settings. They add no material value when the outcome is defined from the same activity indicators used by the Baseline model.

### 4.7 Experimental analysis

#### 4.7.1 Generalization analysis

Robustness was examined in three ways. First, the survey model was compared across item, parcel-latent, parcel-scale, and ordered-item sensitivity specifications. Second, the text analysis used a separate Copilot context and excluded blank responses. Third, DTA prediction used an untouched test set, a descriptive repeated analysis, and 30%–50% course-calendar windows. These checks assess sensitivity within modules; they do not establish participant-level or contextual generalization across the independent datasets.

The survey direct paths TA → RU, RU → PSE, TA → ENG, and RU → ENG were consistent with the theoretical rationale, but the indirect effects were not significant. In the Copilot analysis, neither the TA → *RUI*^*text*^ path nor the text-based indirect effect was significant. DTA recovery features produced a small held-out difference and negligible average window-sensitivity differences. RAIAF is therefore a cautious organization of complementary evidence, not a universal mediation model.

#### 4.7.2 Explanation analysis

Permutation importance was used to describe the behavioral trace models. For activity-defined engagement prediction, the most important features were early active days, early active weeks, and early unique tasks solved, reflecting the construction of the activity-based outcome. The ranked permutation-importance features are reported in Table 12.

**Table 12:** Top permutation-importance features for DTA prediction.

Target	Feature	Importance mean
*activity*_*defined*_*engagement*	early_active_days	0.066
	early_active_weeks	0.047
	early_unique_tasks_solved	0.016
	early_error_after_success_count	0.012
	early_total_attempts	0.006
*final*_*success*_*all*_11	early_unique_tasks_solved	0.016
	early_unique_tasks_attempted	0.009
	early_study_span_days	0.005
	early_accepted_count	0.004
	early_success_after_failure	0.003

For final-success prediction, the most important features were early unique tasks solved, early unique tasks attempted, early study span days, early accepted count, and early success after failure. The last variable is compatible with recovery after error, but permutation importance cannot establish reflection, mechanism, or causality. Figure 4 visualizes these small predictive contributions.

#### 4.7.3 Parameter analysis

The main behavioral parameter is the common course-calendar window. The primary 40% cutoff was selected as an intermediate descriptive point between earlier detection and accumulation of sufficient events. Table 13 reports 30%, 40%, and 50% sensitivity results using the locked learner specifications. More students become observable as the window expands, but the reflective-feature Macro-F1 difference remains between –0.001 and 0.002. Thus, the held-out 0.018 difference should not be generalized as a stable practical gain.

**Table 13:** Calendar-window sensitivity using repeated five-fold evaluation with locked learner specifications.

Target	Window	*N*	Baseline	+ Reflective	Difference
Final success	30%	1,085	0.691	0.692	0.000
	40%	1,122	0.698	0.699	0.001
	50%	1,152	0.680	0.680	0.000
Activity-defined engagement	30%	1,085	0.772	0.772	< −0.001
	40%	1,122	0.783	0.782	–0.001
	50%	1,152	0.815	0.814	–0.001

Values are mean Macro-F1.

The second design parameter is the activity-defined engagement threshold. The 67th percentile avoids an overly rare positive class, but its choice is conventional and the resulting label overlaps with activity predictors.

The third design choice is feature fusion. Recovery-related features produced slightly larger held-out differences than the SE-related group for final success, but neither augmented group showed a stable practical advantage. The Full Model did not consistently outperform simpler configurations.

The fourth design choice is the model family. RAIAF is not a neural-backbone method. It uses conventional classifiers to keep the behavioral trace pipeline interpretable. Therefore, parameter analysis focuses on early-window size, label construction, and feature-group design rather than neural backbone comparison.

#### 4.7.4 Limitation

Several limitations should be acknowledged. First, the survey sample was fixed at *N* = 131 and was likely underpowered for small indirect effects. Parceling substantially improved fit over the item-level MLR model and can conceal item-level misspecification. Although parcels followed predefined subdimensions and their loadings were strong, item-level CFI and TLI were weak, TA–ENG HTMT exceeded 0.85, and the item-level WLSMV sensitivity solution was inadmissible. The parcel results therefore require replication with a prospectively powered sample and a validated measurement model. Second, none of the indirect effects was significant. The contribution is limited to selected direct associations, including TA → RU, RU → PSE, TA → ENG, and RU → ENG, rather than a serial mediation process.

Third, the Copilot text analysis remains exploratory despite the addition of independent double human coding. Exact agreement was 57.6%, linear-weighted Cohen's κ was 0.421, and Krippendorff's ordinal α was 0.495. These estimates indicate limited initial agreement, especially at the boundaries between adjacent categories, and do not justify treating *RUI*^*text*^ as a fully validated scale. Disagreements were resolved by consensus for analysis, but consensus does not replace the initial reliability evidence. In addition, coding was conducted from Chinese translations of the English responses; although programming tokens were preserved, translation may have reduced some semantic nuance. Future studies should refine and pilot the category boundaries before full coding and validate the measure in larger multilingual samples. Fourth, the Copilot technology-acceptance measure shows a specific ceiling effect: 28.9% of participants received the maximum score and 81.6% scored at least 4.5 on the five-point scale. This restricted variance likely attenuated associations involving technology acceptance and may have contributed to the non-significant text-based paths.

Fifth, DTA does not measure AI use, reflection, or self-efficacy directly; its recovery and persistence variables are behavioral approximations from a different programming context. Sixth, the activity-defined engagement label overlaps with baseline predictors and does not provide an independent psychological criterion. Seventh, the held-out predictive differences are small, and the window-sensitivity differences are negligible; no practical intervention benefit has been demonstrated.

Finally, the study is based on secondary datasets. Although this design improves reproducibility, it limits control over task design, measurement timing, and intervention conditions. The DTA trace dataset is especially useful for examining programming behavior, but it should not be interpreted as a direct observation of AI-assistant use. Future research should collect longitudinal data that combine real-time AI interactions, validated self-efficacy scales, process-level reflection measures, and experimental or quasi-experimental reflection prompts.

## 5 Conclusions

This study examined reflective AI use as an educational-psychological issue in AI-supported programming. The survey and Copilot modules addressed AI-supported contexts, while the independent DTA module supplied complementary programming-behavior evidence only. In the survey, technology acceptance predicted reflective use and engagement, and reflective use predicted programming self-efficacy and engagement. The indirect effects were not significant, and the text results did not replicate the proposed paths. The proposed mechanism was therefore only partially supported.

The behavioral trace results were more limited. Recovery-related features produced a small held-out difference for final completion, but repeated and calendar-window analyses showed negligible average gains. For activity-defined engagement, baseline activity features were already strong because the label used overlapping activity indicators. These results do not demonstrate practical educational benefit or validate reflective AI use.

Future work should extend this study in three directions. First, longitudinal data should be collected to examine how students' acceptance, reflection, and self-efficacy change over time. Second, future studies should include richer AI interaction logs, such as prompts, generated code, revision traces, and feedback responses. Third, text-based reflective-use coding should be improved through pilot calibration, clearer boundary rules, independent multilingual coding, and stronger intercoder reliability. Experimental or quasi-experimental designs could also test whether reflection prompts embedded in AI programming tools can strengthen students' reflective use and improve later learning outcomes.

Overall, this study shows that AI-supported programming learning should not be evaluated only by whether students accept or frequently use AI tools. A more useful perspective is to examine how students respond to AI support, whether they revise and learn from feedback, and when such behaviors improve learning outcomes. RAIAF provides a transparent and reproducible step toward connecting educational-psychological constructs with behavioral trace evidence.

## Data Availability

The datasets analyzed in this study are publicly available. The AI-supported programming survey dataset is available from Mendeley Data (doi: 10.17632/bmg4r393zw.1). The GitHub Copilot field-study dataset is available from Mendeley Data (doi: 10.17632/vmzrd2rjgj.2). The DTA programming dataset is available from Zenodo (doi: 10.5281/zenodo.7799972) and is described in Demidova et al. (2023).

## References

[B1] Azevedo R. Witherspoon A. M. Chauncey A. Burkett C. Fike A. (2009). “Metatutor: a metacognitive tool for enhancing self-regulated learning,” in Proceedings of the AAAI fall symposium on cognitive and metacognitive educational systems (Arlington, VA), 14–19.

[B2] Aziz N. (2026). Github Copilot in Computing Education: A Field Study Dataset. Amsterdam: Mendeley Data. doi: 10.17632/vmzrd2rjgj.2

[B3] Baker R. S. J. d. Yacef K. (2009). The state of educational data mining in 2009: a review and future visions. J. Educ. Data Min. 1, 3–17. doi: 10.5281/zenodo.3554657

[B4] Bandura A. (1997). Self-Efficacy: The Exercise of Control. New York, NY: W. H. Freeman.

[B5] Becker B. A. Denny P. Finnie-Ansley J. Luxton-Reilly A. Prather J. Santos E. A. (2023). “Programming is hard-or at least it used to be: educational opportunities and challenges of AI code generation,” in Proceedings of the 54th ACM technical symposium on computer science education (Toronto, ON), 500–506. doi: 10.1145/3545945.3569759

[B6] Davis F. D. (1989). Perceived usefulness, perceived ease of use, and user acceptance of information technology. MIS Q. 13, 319–340. doi: 10.2307/249008

[B7] Demidova L. A. Andrianova E. G. Sovietov P. N. Gorchakov A. V. (2023). Dataset of program source codes solving unique programming exercises generated by digital teaching assistant. Data 8:109. doi: 10.3390/data8060109

[B8] Denny P. Prather J. Becker B. A. Finnie-Ansley J. Hellas A. Leinonen J. . (2024). Computing education in the era of generative AI. Commun. ACM 67, 56–67. doi: 10.1145/3624720

[B9] Doshi A. R. Hauser O. P. (2024). Generative ai enhances individual creativity but reduces the collective diversity of novel content. Sci. Adv. 10:eadn5290. doi: 10.1126/sciadv.adn5290 PMC11244532 38996021

[B10] Fredricks J. A. Blumenfeld P. C. Paris A. H. (2004). School engagement: potential of the concept, state of the evidence. Rev. Educ. Res. 74, 59–109. doi: 10.3102/00346543074001059

[B11] Fritz M. S. MacKinnon D. P. (2007). Required sample size to detect the mediated effect. Psychol. Sci. 18, 233–239. doi: 10.1111/j.1467-9280.2007.01882.x PMC2843527 17444920

[B12] Gasevic D. Dawson S. Siemens G. (2015). Let's not forget: learning analytics are about learning. Techtrends 59, 64–71. doi: 10.1007/s11528-014-0822-x

[B13] Gunasekara S. Saarela M. (2024). “Explainability in educational data mining and learning analytics: an umbrella review,” in Proceedings of the 17th international conference on educational data mining (Atlanta, GA), 887–892

[B14] Henseler J. Ringle C. M. Sarstedt M. (2015). A new criterion for assessing discriminant validity in variance-based structural equation modeling. J. Acad. Mark. Sci. 43, 115–135. doi: 10.1007/s11747-014-0403-8

[B15] Holmes W. Bialik M. Fadel C. (2019). Artificial Intelligence in Education: Promises and Implications for Teaching and Learning. Boston, MA: Center for Curriculum Redesign.

[B16] Hoq M. Brusilovsky P. Akram B. (2023). “Analysis of an explainable student performance prediction model in an introductory programming course,” in Proceedings of the 16th international conference on educational data mining (Bengaluru), 79–90.

[B17] Islam M. M. Sojib F. H. Mihad M. F. H. Hasan M. Rahman M. (2025). The integration of explainable AI in educational data mining for student academic performance prediction and support system. Telemat. Inform. Rep. 18:100203. doi: 10.1016/j.teler.2025.100203

[B18] Kasneci E. Sessler K. Küchemann S. Bannert M. Dementieva D. Fischer F. . (2023). Chatgpt for good? On opportunities and challenges of large language models for education. Learn. Individ. Differ. 103:102274. doi: 10.1016/j.lindif.2023.102274

[B19] Khosravi H. Shum S. B. Chen G. Conati C. Tsai Y.-S. Kay J. . (2022). Explainable artificial intelligence in education. Comput. Educ. Artif. Intell. 3:100074. doi: 10.1016/j.caeai.2022.100074

[B20] Little T. D. Cunningham W. A. Shahar G. Widaman K. F. (2002). To parcel or not to parcel: exploring the question, weighing the merits. Struct. Equ. Model. 9, 151–173. doi: 10.1207/S15328007SEM0902_1

[B21] Long P. Siemens G. (2011). Penetrating the fog: analytics in learning and education. Educause Rev. 46, 30–40. doi: 10.17471/2499-4324/195

[B22] Luckin R. Holmes W. Griffiths M. Forcier L. B. (2016). Intelligence Unleashed: An Argument for AI in Education. London: Pearson.

[B23] Lundberg S. M. Lee S.-I. (2017). A unified approach to interpreting model predictions. Adv. Neural Inf. Process. Syst. 30, 4765–4774. doi: 10.48550/arXiv.1705.07874

[B24] Matsunaga M. (2008). Item parceling in structural equation modeling: a primer. Commun. Methods Meas. 2, 260–293. doi: 10.1080/19312450802458935

[B25] Milano S. McGrane J. A. Leonelli S. (2023). Large language models challenge the future of higher education. Nat. Mach. Intell. 5, 333–334. doi: 10.1038/s42256-023-00644-2

[B26] Miller T. (2019). Explanation in artificial intelligence: insights from the social sciences. Artif. Intell. 267, 1–38. doi: 10.1016/j.artint.2018.07.007

[B27] Nathaniel J. Oyelere S. S. Suhonen J. Tedre M. (2025). Literature review on the integration of generative ai in programming education. Int. J. Artif. Intell. Educ. 35, 2724–2755. doi: 10.1007/s40593-025-00524-3

[B28] Nicol D. J. Macfarlane-Dick D. (2006). Formative assessment and self-regulated learning: a model and seven principles of good feedback practice. Stud. High. Educ. 31, 199–218. doi: 10.1080/03075070600572090

[B29] Noy S. Zhang W. (2023). Experimental evidence on the productivity effects of generative artificial intelligence. Science 381, 187–192. doi: 10.1126/science.adh2586 37440646

[B30] Ouyang F. Jiao P. (2021). Artificial intelligence in education: the three paradigms. Comp. Educ. Artif. Intell. 2:100020. doi: 10.1016/j.caeai.2021.100020

[B31] Pintrich P. R. (2000). “The role of goal orientation in self-regulated learning,” in Handbook of Self-Regulation, eds. Boekaerts M. Pintrich P. R. Zeidner M. (San Diego, CA: Academic Press), 451–502. doi: 10.1016/B978-012109890-2/50043-3

[B32] Prather J. Denny P. Leinonen J. Becker B. A. Albluwi I. Craig M. . (2023). “The robots are here: navigating the generative ai revolution in computing education,” in Proceedings of the 2023 working group reports on innovation and technology (Turku), 108–159. doi: 10.1145/3623762.3633499

[B33] Raihan N. Siddiq M. L. Santos J. C. S. Zampieri M. (2025). “Large language models in computer science education: a systematic literature review,” in Proceedings of the 56th ACM technical symposium on computer science education V. 1 (Pittsburgh, PA), 938–944. doi: 10.1145/3641554.3701863

[B34] Ramalingam V. Wiedenbeck S. (1998). Development and validation of scores on a computer programming self-efficacy scale and group analyses of novice programmer self-efficacy. J. Educ. Comp. Res. 19, 367–381. doi: 10.2190/C670-Y3C8-LTJ1-CT3P

[B35] Rhemtulla M. Brosseau-Liard P. É. Savalei V. (2012). When can categorical variables be treated as continuous? A comparison of robust continuous and categorical SEM estimation methods under suboptimal conditions. Psychol. Methods 17, 354–373. doi: 10.1037/a0029315 22799625

[B36] Ribeiro M. T. Singh S. Guestrin C. (2016). “Why should I trust you? Explaining the predictions of any classifier,” in Proceedings of the 22nd ACM SIGKDD international conference on knowledge discovery and data mining (San Francisco, CA), 1135–1144. doi: 10.1145/2939672.2939778

[B37] Romero C. Ventura S. (2010). Educational data mining: a review of the state of the art. IEEE Trans. Syst. Man Cybernet. C 40, 601–618. doi: 10.1109/TSMCC.2010.2053532

[B38] Rudin C. (2019). Stop explaining black box machine learning models for high stakes decisions and use interpretable models instead. Nat. Mach. Intell. 1, 206–215. doi: 10.1038/s42256-019-0048-x PMC9122117 35603010

[B39] Shi Y. Yu K. Dong Y. Chen F. (2026). Large language models in education: a systematic review of empirical applications, benefits, and challenges. Comp. Educ. Artif. Intell. 10:100529. doi: 10.1016/j.caeai.2025.100529

[B40] Shihab M. I. H. Hundhausen C. Tariq A. Haque S. Qiao Y. Mulanda B. W. (2025). “The effects of github copilot on computing students' programming effectiveness, efficiency, and processes in brownfield coding tasks,” in Proceedings of the 2025 ACM conference on international computing education research V. 1 (Charlottesville, VA), 407–420. doi: 10.1145/3702652.3744219

[B41] Shute V. J. (2008). Focus on formative feedback. Rev. Educ. Res. 78, 153–189. doi: 10.3102/0034654307313795

[B42] Tang B. Luo H. Chen M. Zhao X. (2026). Dataset on Technology Acceptance, Learning Engagement, Reflective Use, Learning Interest, and Programming Self-Efficacy in AI-Supported Programming Learning. Amsterdam: Mendeley Data. doi: 10.17632/bmg4r393zw.1

[B43] Thorp H. H. (2023). ChatGpt is fun, but not an author. Science 379:313. doi: 10.1126/science.adg7879 36701446

[B44] Venkatesh V. Morris M. G. Davis G. B. Davis F. D. (2003). User acceptance of information technology: toward a unified view. MIS Q. 27, 425–478. doi: 10.2307/30036540

[B45] Winne P. H. Hadwin A. F. (1998). “Studying as self-regulated learning,” in Metacognition in Educational Theory and Practice, eds. Hacker D. J. Dunlosky J. Graesser A. C. (Mahwah, NJ: Lawrence Erlbaum Associates), 277–304.

[B46] Wolf E. J. Harrington K. M. Clark S. L. Miller M. W. (2013). Sample size requirements for structural equation models: an evaluation of power, bias, and solution propriety. Educ. Psychol. Meas. 73, 913–934. doi: 10.1177/0013164413495237 PMC4334479 25705052

[B47] Xie B. Lim J. O. Pham P. K. D. Li M. Ko A. J. (2023). “Developing novice programmers' self-regulation skills with code replays,” in Proceedings of the 2023 ACM conference on international computing education research V. 1 (Chicago, IL), 298–313. doi: 10.1145/3568813.3600127

[B48] Yan L. Sha L. Zhao L. Li Y. Martinez-Maldonado R. Chen G. . (2024). Practical and ethical challenges of large language models in education: a systematic scoping review. Br. J. Educ. Technol. 55, 90–112. doi: 10.1111/bjet.13370

[B49] Zawacki-Richter O. Marin V. I. Bond M. Gouverneur F. (2019). Systematic review of research on artificial intelligence applications in higher education-where are the educators? Int. J. Educ. Technol. High. Educ. 16:39. doi: 10.1186/s41239-019-0171-0

[B50] Zhu M. Xu L. Ericson B. (2025). A systematic review of research on large language models for computer programming education. arXiv [Preprint]. *arXiv:2506.21818*. Available online at: https://arxiv.org/abs/2506.21818 (Accessed August 29, 2026).

[B51] Zimmerman B. J. (2002). Becoming a self-regulated learner: an overview. Theory Pract. 41, 64–70. doi: 10.1207/s15430421tip4102_2

